# Deep learning based automated epidermal growth factor receptor and anaplastic lymphoma kinase status prediction of brain metastasis in non-small cell lung cancer

**DOI:** 10.37349/etat.2023.00158

**Published:** 2023-08-30

**Authors:** Abhishek Mahajan, Gurukrishna B, Shweta Wadhwa, Ujjwal Agarwal, Ujjwal Baid, Sanjay Talbar, Amit Kumar Janu, Vijay Patil, Vanita Noronha, Naveen Mummudi, Anil Tibdewal, JP Agarwal, Subash Yadav, Rajiv Kumar Kaushal, Ameya Puranik, Nilendu Purandare, Kumar Prabhash

**Affiliations:** University of Toronto, Canada; ^1^Clatterbridge Centre for Oncology NHS Foundation Trust, L7 8YA Liverpool, UK; ^2^Department of Radiodiagnosis, Tata Memorial Hospital, Parel, Mumbai 400012, Maharashtra, India; ^3^Department of Electronics and Telecommunication Engineering, SGGS Institute of Engineering and Technology, Nanded 431606, Maharashtra, India; ^4^Department of Medical Oncology, Tata Memorial Hospital, Parel, Mumbai 400012, Maharashtra, India; ^5^Department of Radiation Oncology, Tata Memorial Hospital, Parel, Mumbai 400012, Maharashtra, India; ^6^Department of Pathology, Tata Memorial Hospital, Parel, Mumbai 400012, Maharashtra, India; ^7^Department of Nuclear Medicine, Tata Memorial Hospital, Parel, Mumbai 400012, Maharashtra, India

**Keywords:** Non-small cell lung cancer, epidermal growth factor receptor, anaplastic lymphoma kinase, semantics, radiomics, deep learning, machine learning, convolutional neural networks

## Abstract

**Aim:**

The aim of this study was to investigate the feasibility of developing a deep learning (DL) algorithm for classifying brain metastases from non-small cell lung cancer (NSCLC) into epidermal growth factor receptor (*EGFR*) mutation and anaplastic lymphoma kinase (*ALK*) rearrangement groups and to compare the accuracy with classification based on semantic features on imaging.

**Methods:**

Data set of 117 patients was analysed from 2014 to 2018 out of which 33 patients were *EGFR* positive, 43 patients were *ALK* positive and 41 patients were negative for either mutation. Convolutional neural network (CNN) architecture efficient net was used to study the accuracy of classification using T1 weighted (T1W) magnetic resonance imaging (MRI) sequence, T2 weighted (T2W) MRI sequence, T1W post contrast (T1post) MRI sequence, fluid attenuated inversion recovery (FLAIR) MRI sequences. The dataset was divided into 80% training and 20% testing. The associations between mutation status and semantic features, specifically sex, smoking history, *EGFR *mutation and *ALK *rearrangement status, extracranial metastasis, performance status and imaging variables of brain metastasis were analysed using descriptive analysis [chi-square test (χ^2^)], univariate and multivariate logistic regression analysis assuming 95% confidence interval (CI).

**Results:**

In this study of 117 patients, the analysis by semantic method showed 79.2% of the patients belonged to *ALK* positive were non-smokers as compared to double negative groups (*P* = 0.03). There was a 10-fold increase in *ALK* positivity as compared to *EGFR* positivity in ring enhancing lesions patients (*P* = 0.015) and there was also a 6.4-fold increase in *ALK *positivity as compared to double negative groups in meningeal involvement patients (*P* = 0.004). Using CNN Efficient Net DL model, the study achieved 76% accuracy in classifying *ALK* rearrangement and *EGFR* mutations without manual segmentation of metastatic lesions. Analysis of the manually segmented dataset resulted in improved accuracy of 89% through this model.

**Conclusions:**

Both semantic features and DL model showed comparable accuracy in classifying *EGFR *mutation and *ALK *rearrangement. Both methods can be clinically used to predict mutation status while biopsy or genetic testing is undertaken.

## Introduction

Lung cancer is one of the major challenges in medical oncology. It is the most common cause of cancer related death in the world [1–3]. Up to 64% of all patients with lung cancer develop brain metastasis [4, 5]. Data from several patient series have shown that the median age of anaplastic lymphoma kinase (*ALK*) positive non-small cell lung cancer (NSCLC) patients is 55 years which is about 10 years to 15 years lower than for the general NSCLC population and also the epidermal growth factor receptor (*EGFR*) mutated patients, approximately 70% of these patients are never smokers [6]. Human genome sequencing has ensured identification of epigenetic mutations, tumour-suppressor-gene inactivation and pro-oncogene mutations that might be potentially targeted for therapy [7–12]. For NSCLC, *EGFR* mutations and on abnormal fusion of *ALK* being targeted successfully with *EGFR* tyrosine kinase inhibitors (TKI) and ALK inhibitors respectively.

Classically, brain metastases seen on magnetic resonance imaging (MRI) occurring at grey and white matter junction can have varying imaging parameters such as size, post contrast enhancement, peri-lesional edema. There has been increased interest in research for the characterization of quantitative imaging features reflecting tumor biology, physiology and tumor phenotype using artificial intelligence (AI) based algorithms. Radiomics and deep learning (DL)-AI based models are extensively used in medical imaging [13–15] which basically involves image segmentation and detection. This image segmentation usually relies on manual delineation which is time-consuming and subject to inter- or intra-segmentation variation. Secondly, despite the segmentation of images being accurate, no standard method for image feature extraction is available and errors due to miscalculation are encountered as it is difficult to verify the accuracy and reproducibility of image features.

Advanced AI models such as the neural network based DL method can overcome these problems through a self-learning strategy and presents a promising tool for genomic analysis [16–19]. The DL method can be correlated with the functioning of the neural network in the brain. In comparison to radiomic methods, precise tumor boundary annotation is not required with DL and thus saves a lot of time and human effort [20–24]. Furthermore, DL method takes into consideration the microenvironment of the surrounding lung parenchyma and can extract features that are adaptive to specific clinical outcomes, whereas radiomics can only describe general features which lacks specificity for outcome prediction [25–29].

The primary objective of this study was to develop a novel DL model to segment brain metastasis and to categorize the lesions into *EGFR* positive, *ALK* positive and double negative groups. Additionally, classification was also performed based on semantic features i.e., clinical and imaging features.

## Materials and methods

### Patient cohort

Between January 2014 and December 2018, there were 117 lung cancer patients with brain metastasis underwent molecular profiling, from which 33 patients were *EGFR* positive, 43 patients were *ALK* positive and 41 patients were negative for either mutation. DL-based classification data was highly imbalanced as predominant sample size belongs to adenocarcinoma class and only a small proportion of cases belonged to squamous carcinoma groups. Hence, the classification was based on presence and absence of the *EGFR* mutation and *ALK* rearrangement. These patients underwent pre-treatment MRI brain at Tata Memorial Hospital (TMH), had the necessary data in digital imaging and communications in medicine (DICOM) format on picture archiving and communication system (PACS) and clinical data on endoscopic mucosal resection (EMR), and were therefore included in this study. Cases lacking molecular profiling and those who underwent local therapy were excluded from this study. Patients on systemic chemotherapy were included in study. This study was conducted under project number 3296 in TMH institutional ethics committee (IEC-1).

### Radiology review

The DL model was trained using one or more slices containing the tumour and optimized to use the slice with the maximum dimension of the tumour. For classification of semantic features, a clinical radiologist in TMH with 10 years of experience in MRI of brain tumours and other radiologists with 2 years’ experience retrospectively interpreted the magnetic resonance (MR) images independently. Both radiologists were blinded to clinical and histologic findings.

### Methodology

#### Classification using semantic features

MRI was performed on two 1.5 Tesla MRI scanners–General Electric (GE), Signa HDxt 1.5 T (Milwaukee, Wisconsin, USA) and Philips Ingenia 1.5 Tesla (Amsterdam, The Netherlands and one 3.0 Tesla MRI scanner–GE, Signa HDxt 3.0 Tesla (Milwaukee, Wisconsin, USA). Clinical parameters assessed were sex, smoking history, *EGFR* mutation status, *ALK* rearrangement status, histological subtype, extracranial metastasis, performance status, treatment received, response to therapy, date of diagnosis, date of detection of metastasis and time from diagnosis to response to treatment, as shown in Table S1.

#### Development of DL algorithm and classification

**Figure 1:**
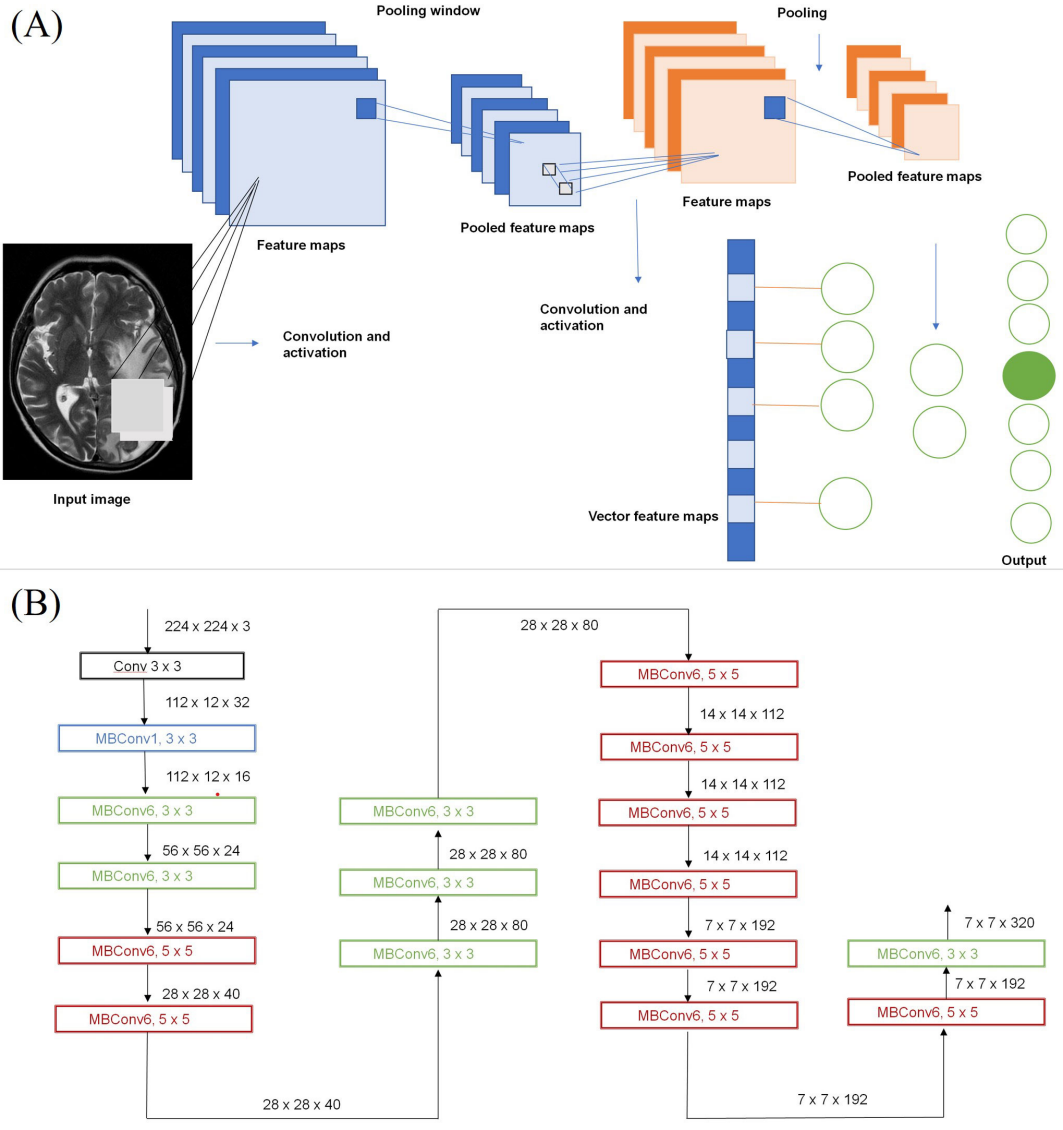
Structure of CNN. (a) Building blocks of a typical CNN; (b) architecture for baseline network EfficientNetB0

**Figure 2:**
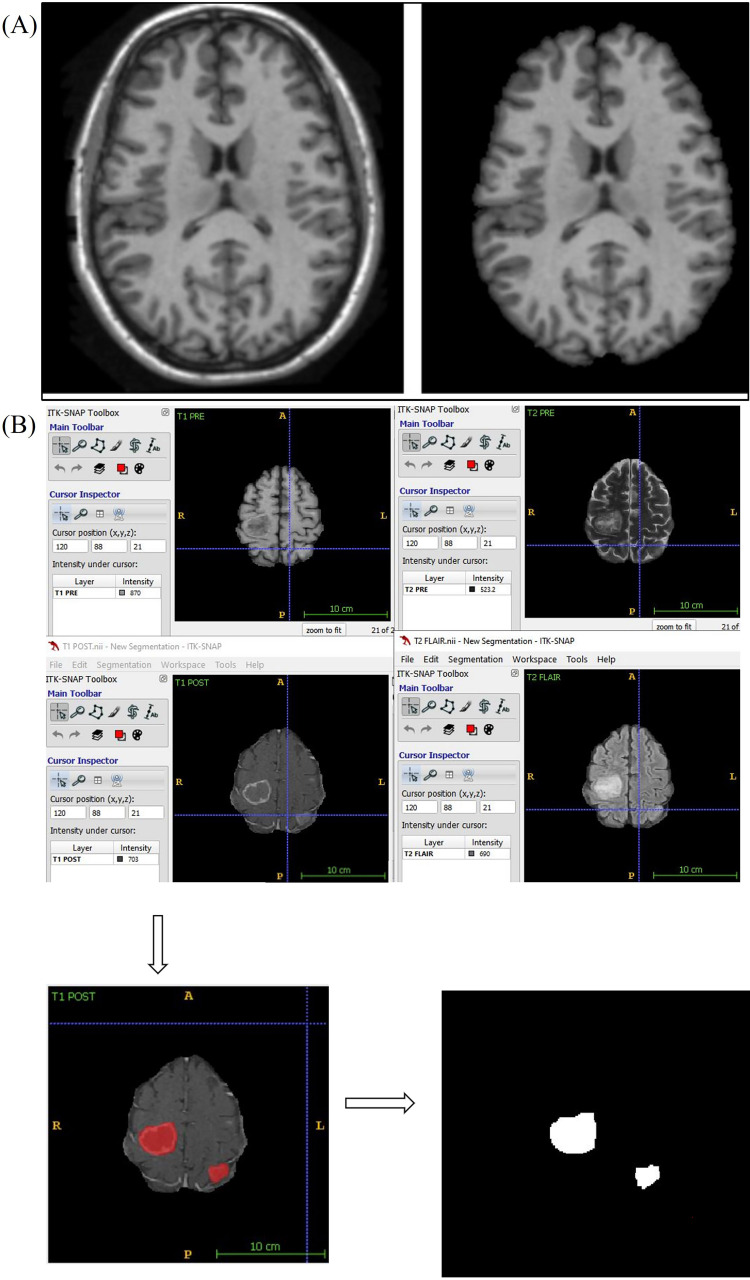
Skull stripping and annotation of images. (a) Axial T1W MRI sequence image the brain before and after skull stripping; (b) annotation of MR images. A: anterior; P: posterior; R: right; L: Left

Using a convoluted neural network, DL aims to learn the abstract mapping between raw data to the desired label. The main components of convolutional neural networks (CNN) are convolution, pooling, activation and batch normalization (refer to Figure 1A). EfficientNetB0 CNN architecture uses a compound scaling method, which systematically improves model performance by balancing all compound coefficients of the architecture width, depth, and image resolution (Figure 1B). The overall dataset is divided into three main categories namely, *EGFR* positive (33 cases), *ALK* positive (43 cases) and double negative (41 cases). For the classification of MR images, each case is provided with four MR sequences: T1, T2, T1 weighted (T1W) post contrast (T1post), and fluid attenuated inversion recovery (FLAIR). Firstly, the data was converted from DICOM to Neuroimaging Informatics Technology Initiative (NIfTI) format and all four MR sequences were skull stripped and brain volume was extracted. Further, the MR images were co-registered and interpolated to 1 mm^3^ resolution (refer to Figure 2A). Metastases for all the cases were then annotated manually using ITK snap toolbox (Refer to Figure 2B). The annotated MR dataset was then classified into three classes with popular DL-based classifiers with transfer learning and performance. Heavy data augmentation was used to increase the data for training in which the dataset was divided into 80% training and 20% test dataset. Efficient Net B0 model was implemented with Keras code which is based on the Keras package with tensor flow as backend in Python. Keras makes use of graphical processing units (GPUs) to speed up the DL algorithms. Training was done using CNN architecture on an NVIDIA P100 graphics processing unit (GPU) card with 16 giga byte (GB) memory which took about 32 h.

#### Statistical analysis

Statistical analysis was performed using Statistical Package for Social Sciences (IBMSPSS; IBM Corp., Armonk, NY, USA) version 21. Data was descriptively analysed using frequency and percentage for categorical data and mean [standard deviation (SD)]/median [inter quartile range (IQR)] for continuous data. Chi-square test (χ^2^) for independence was used to compare association between two categorical variables. Continuous data was compared using independent *t* test/Mann-Whitney *U* test as appropriate. A multivariable multinomial logistic regression was performed to model the relationship between the predictors of mutation in the three groups (double negative, *EGFR* positive, *ALK* positive). Area under the curve (AUC) and receiver operating characteristics (ROC) curves were used to present the accuracy of different predictive models. All statistics were 2-sided, and a value of *P* < 0.05 was considered statistically significant.

## Results

### Results of classification based on clinical and MRI semantic features

**Figure 3:**
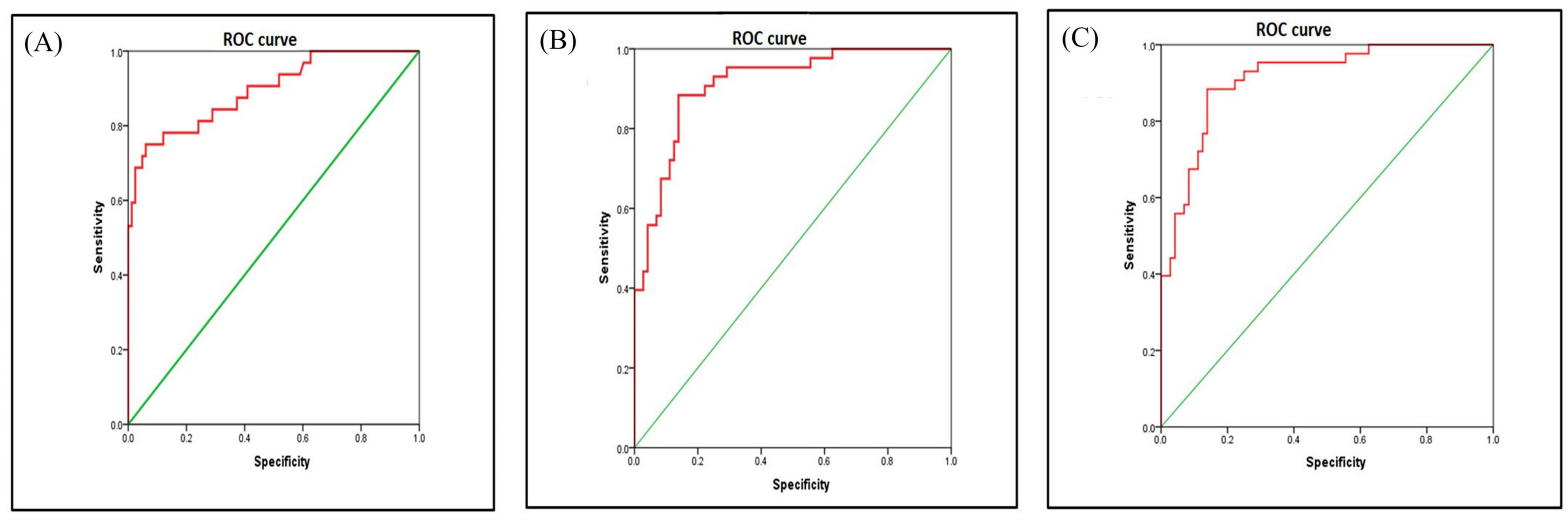
ROC of dataset. (a) ROC areas of *EGFR* mutation *vs.* negative; (b) ROC areas of *ALK* mutation *vs.* negative; (c) ROC areas of *ALK* positive *vs. EGFR* mutation

For statistical analysis using the semantic method, association between the mutation groups and clinical parameters were assessed using χ^2^. The dataset was divided into *EGFR* mutation positive, *ALK* rearrangement positive and double negative groups. Multivariate analysis was performed between *EGFR* positive and double negative group, *ALK* positive and double negative group (Table 1) and *ALK* positive and *EGFR* positive groups (Table 2). Statistical results from revealed that lesions showing fuzzy T2 borders and intralesional haemorrhage had 7.8 times and 6.5 times more odds of being *EGFR* positive respectively (*P* = 0.009 and 0.025, respectively) as opposed to double negative group with an AUC of 0.894 [95% confidence interval (CI): 0.825–0.964] (Figure 3A).

**Table 1:** Multivariate analysis of EGFR and ALK positive *vs.* double negative groups

Predictor variables	Level	EGFR positive *vs.* negative		ALK positive *vs.* negative	
		**Sig.**	**OR**	**Sig.**	**OR**
Intercept	-	0.691	-	0.618	-
Number of lesions	-	0.061	1.028 (0.999, 1.057)	0.013	1.037 (1.008, 1.068)
Smoking history	Yes	0.147	0.316 (0.066, 1.499)	0.037	0.208 (0.047, 0.913)
	No	REF			
Histology	Squamous	0.826	0.769 (0.074, 7.949)	0.164	0.160 (0.012, 2.112)
	Adenocarcinoma	REF			
T2W	Hypointense	0.413	0.424 (0.054, 3.311)	0.123	4.099 (0.684, 24.573)
	Heterogeneous	0.707	0.630 (0.057, 6.977)	0.467	0.360 (0.023, 5.661)
	Hyperintense	REF			
T2 borders	Defined	REF			
	Fuzzy	0.009	7.858 (1.681, 36.742)	0.242	2.401 (0.554, 10.408)
Hemorrhage	Present	0.025	6.490 (1.267, 33.240)	0.771	0.787 (0.157, 3.952)
	Absent	REF			
Diffusion restriction	Central	0.302	0.329 (0.040, 2.721)	0.479	0.402 (0.032, 4.994)
	Complete	0.058	0.100 (0.009, 1.080)	0.106	0.113 (0.008, 1.586)
	Peripheral	0.023	0.044 (0.003, 0.650)	0.074	4.227 (0.867, 20.600)
	None	REF			
Enhancement pattern	Patchy	0.198	3.606 (0.51, 25.478)	0.292	2.946 (0.394, 22.004)
	Ring	0.715	1.43 (0.209, 9.775)	0.005	14.879 (2.278, 97.189)
	Homogeneous	REF			
Meningeal involvement	Present	0.594	1.584 (0.292, 8.591)	0.045	6.394 (1.046, 39.071)
	Absent	REF			
Lung metastasis	Present	0.054	6.329 (0.967, 41.427)	0.007	12.47 (1.995, 77.925)
	Absent	REF			

Sig.: significance; OR: odds ratio; -: not applicable; REF: reference; T2W: T2 weighted. Data in parentheses () are 95% CIs

**Table 2:** Multivariate analysis of ALK positive *vs.* EGFR positive groups

**Predictor variables**	**Level**	**Sig.**	**OR**
Intercept	-	0.939	-
Number of lesions	-	0.030	1.009 (1.001, 1.018)
Smoking history	Yes	0.636	0.659 (0.117, 3.705)
	No	REF	
Histology	Squamous	0.358	0.208 (0.007, 5.926)
	Adenocarcinoma	REF	
T2W	Hypointense	0.025	9.668 (1.338, 69.884)
	Heterogeneous	0.705	0.571 (0.031, 10.354)
	Hyperintense	REF	
	Defined	REF	
	Fuzzy	0.113	0.306 (0.071, 1.324)
Hemorrhage	Present	0.013	0.121 (0.023, 0.646)
	Absent	REF	
Diffusion restriction	Central	0.866	1.222 (0.120, 12.458)
	Complete	0.926	1.124 (0.096, 13.167)
	Peripheral	0.001	95.513 (5.946, 1534.295)
	None	REF	
Enhancement pattern	Patchy	0.826	0.817 (0.135, 4.959)
	Ring	0.015	10.404 (1.58, 68.526)
	Homogeneous	REF	
Meningeal involvement	Present	0.124	4.036 (0.682, 23.893)
	Absent	REF	
Lung metastasis	Present	0.372	1.970 (0.445, 8.729)
	Absent	REF	

Data in parentheses () are 95% CIs; -: not applicable

Smokers were 79.2% less likely to be *ALK* positive as opposed to double negative groups (*P* = 0.037). Lesions which showed ring enhancement and meningeal involvement had 14.8 times and 6.4 times more odds of being *ALK* positive respectively (*P* = 0.005 and *P* = 0.045, respectively) as compared to the double negative group. Presence of synchronous lung metastasis was 12.5 times more prevalent in *ALK* positive as compared to double negative group (*P* = 0.007) with an AUC of 0.912 [95% CI: 0.859–0.965] (Figure 3B). Statistical results from (Table 2) revealed that lesions, which showed peripheral diffusion restriction, had 95% more odds of being *ALK* positive as compared to *EFGR* positive group showing no diffusion restriction (*P* = 0.001). Lesions which showed ring enhancement and T2W hypo intense signal intensity had 10 times more odds of being *ALK* positive as compared to *EGFR* positive group (*P* = 0.015 and *P* = 0.025, respectively), with an AUC of 0.912 [95% CI: 0.859–0.965] (Figure 3C).

### Results of DL-based classification

**Figure 4:**
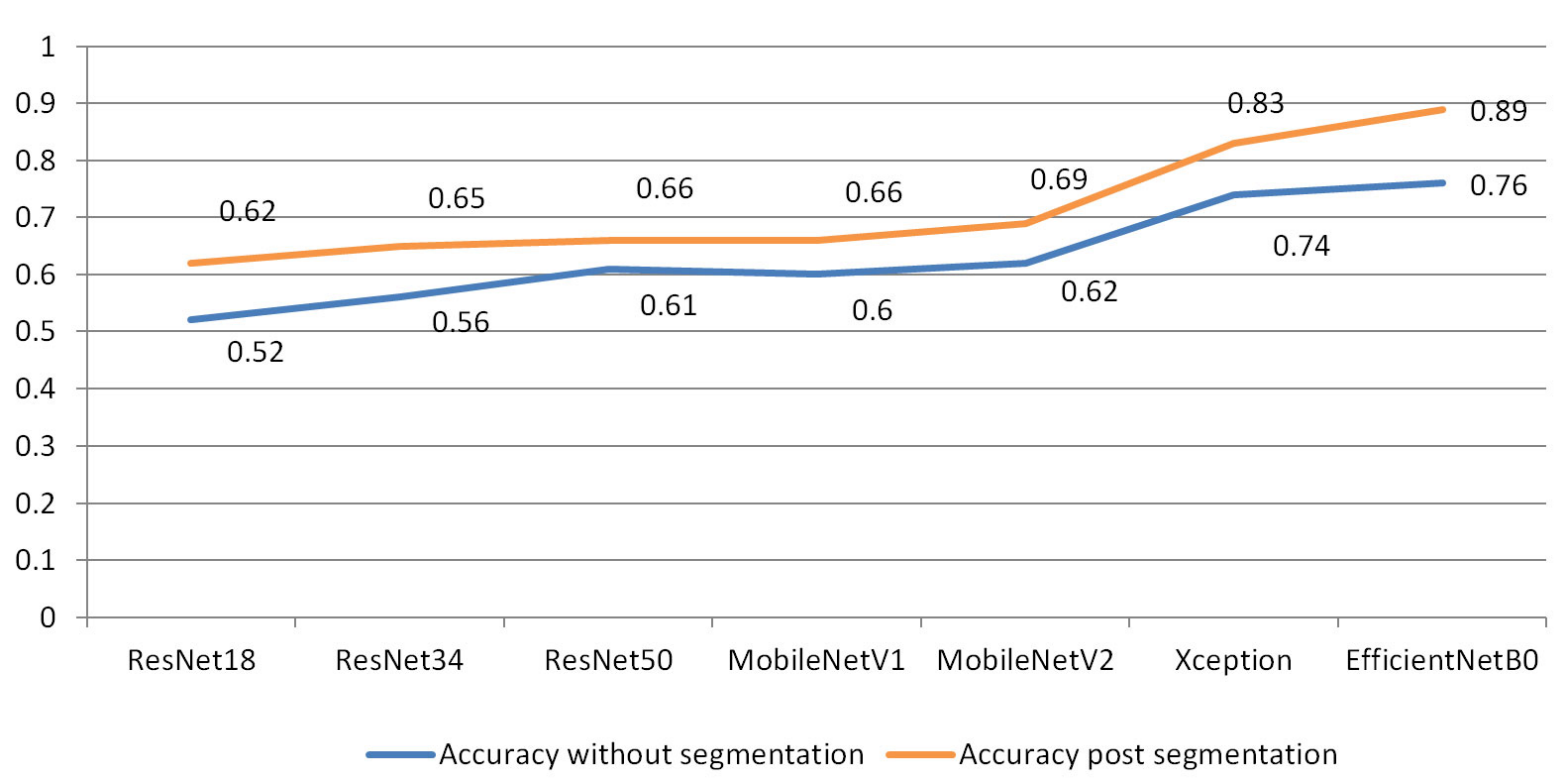
Architecture showing accuracies pre and post segmentation

For analysis using DL models, the dataset was divided into 80% training and 20% testing. As each class had less than 50 patients, the augmented data was reshuffled for the classification task and for unbiased training of the classifier heavy data augmentation techniques. Different deep neural network architectures were used to classify the brain metastatic lesions. EfficientNetB0 showed the greatest accuracy among other CNN architectures in classifying the brain metastasis with an accuracy of 76% prior to manual segmentation of lesions, which got improved post segmentation accuracy of 89% (Table 3 and Figure 4). Accuracy of classification was shown to be significantly higher when aided by manual segmentation across all CNN architectures used in the study.

**Table 3:** Accuracy on validation/hold-out dataset

**Architecture name**	**Accuracy without segmentation**	**Accuracy post segmentation**
ResNet18	0.52	0.62
ResNet34	0.56	0.65
ResNet50	0.61	0.66
MobileNetV1	0.60	0.66
MobileNetV2	0.62	0.69
Xception	0.74	0.83
EfficientNetB0	0.76	0.89

## Discussion

A significant proportion of patients with primary lung cancer develop brain metastasis during the disease course. Recent developments in the research of pathophysiology and molecular biology have led to the further understanding of different molecular mutations, which have been proven potentially useful in therapeutic oncology [30–32]. *EGFR* mutation and *ALK* rearrangement have been in focus recently since they can be targeted for molecular therapy. Recently developed and more specific molecular targeted therapy such as osimertinib in *EGFR* mutation [33] has been proven to increase the survival in patients with a specific mutation.

In the first part of the study, 117 patients were analysed of carcinoma lung with positive MRI scan for brain metastasis in which clinical parameters showed statistical significance. In this study, clinical parameters viz., age and smoking history showed a statistically significant impact on mutation status (*P* = 0.001 and *P* = 0.0002, respectively). A clinical study conducted by Gao et al. [34] showed age and smoking history have a statistically significant impact on *ALK* status. Another clinical study by Bhatt et al. [35] showed age and sex had a statistically significant impact on *EFGR* mutation (*P* = 0.001 and *P* = 0.0001, respectively). Similarly, a study by Rangachari et al. [36] statistically significant impact of age on *EFGR* mutation (*P* = 0.0001). However, it should be noted that above-mentioned studies did not use imaging parameters. Statistical analysis of semantic features in the current study showed both *EGFR* mutation and *ALK* rearrangement were significantly less prevalent in smokers who developed NSCLC. Fuzzy T2 borders and intralesional haemorrhage could be used to differentiate *EGFR* positivity from the double negative group. Ring enhancement, meningeal involvement and synchronous lung metastasis can be used to differentiate *ALK* positivity from the double negative group. Peripheral diffusion restriction, ring enhancement and T2W hypo intense signal intensity can be used to differentiate *ALK* positivity from *EFGR* positivity.

In the second part of the study, performance of the DL model for classification of brain metastases was evaluated. A deeper understanding of radiomics has shown much finer imaging features specific to a mutation can be delineated even when using standard imaging sequences. But consistent manual identification of such features might not always be accurate and is more time consuming. Development of an automated computed algorithm, which can be trained to identify the subtle subclinical imaging features with precision and within a much shorter timeframe, was the purpose of this study. Pre-treatment basic MR sequences namely T1, T2, FLAIR and T1post contrast sequences of 117 patients having brain metastasis belonging to *EGFR* positive, *ALK* positive and double negative groups were included. The case parameters were derived from the studies by Wadhwa et al. [37] and Mahajan et al. [38]. To homogenize the MR images across different sequences, skull stripping, co-registration and brain volume extraction was done. Then all the metastatic brain lesions were manually segmented using ITK snap toolbox. Multiple neural network architectures were fed with both unsegmented and manually segmented datasets. DL-based classification data was highly imbalanced as predominant sample size belongs to adenocarcinoma class and only a small proportion of cases belonged to squamous carcinoma groups. Hence the classification was based on the presence and absence of the *EGFR* mutation and *ALK* rearrangement. For the classification task, each class had less than 50 patients. To train the classifier, heavy data augmentation techniques like rotation, flip, etc. available in Keras were used. The augmented data was reshuffled for unbiased training. This dataset is then divided into 80% training and 20% test dataset. The models were trained to optimize the loss and to improve classification accuracy. DL model showed good predictive performance with pre-segmentation accuracy of 76% and post segmentation accuracy of 89%. The performance of the model in classifying the metastases improved significantly after the manual segmentation. During training and validation, since the case data was shuffled, class-wise true positive (TP), true negative (TN) and false positive (FP), false negative (FN) was not covered in the analysis. EfficientNetB0 with improved model scaling provides better accuracy over other CNN architectures and hence can be used to train complex data sets. No other published study shares similar objectives with this study.

This study concludes that both semantic features and DL model showed comparable accuracy in classifying *EGFR* mutation and *ALK* rearrangement in metastatic brain lesions. Application of both the methods in clinical practice may be useful to predict mutations in a patient while the biopsy or genetic mutation testing is awaited.

This was a site-specific study on NSCLC patients. Hence, the data is not applicable to other primary cancers such as breast or colon cancer. The radiomics and DL model were trained only on brain metastases from adenocarcinoma and a few cases of squamous cell carcinoma of the lung. Other genetic mutation i.e., ROS proto-oncogene 1, receptor tyrosine kinase (*Ros-1)* and Kristen rat sarcoma viral oncogene homolog (*KRAS*) mutations were not included in the current study. As each class had less than 50 patients, the augmented dataset was reshuffled for the classification task and for unbiased training of the classifier’s heavy data augmentation techniques. This dataset was then divided into 80% training dataset and 20% test dataset. Hence the class-wise analysis of true positive, true negative, false positive, and false negative was not feasible in this study. The performance of the proposed approach will significantly improve with a larger dataset in each class for more accurate classification.

In conclusion, both semantic features and DL models showed comparable accuracy in classifying *EGFR* mutation and *ALK* rearrangement. Both methods are non-invasive and can be clinically used to predict mutation status while invasive procedures-biopsy or genetic testing is undertaken. Application of both the methods in future clinical practice may help predict mutations in a patient while the biopsy or genetic mutation testing is awaited.

## Abbreviations

AI: artificial intelligence
ALK: anaplastic lymphoma kinase
AUC: area under the curve
CI: confidence interval
CNN: convolutional neural networks
DL: deep learning
EGFR: epidermal growth factor receptor
MR: magnetic resonance
MRI: magnetic resonance imaging
NSCLC: non-small cell lung cancer
REF: reference
ROC: receiver operating characteristics
T1W: T1 weighted
T2W: T2 weighted
TMH: Tata Memorial Hospital

## Supplementary materials

The supplementary material for this article is available at: https://www.explorationpub.com/uploads/Article/file/1002158_sup_1.pdf.

## Declarations

### Availability of data and materials

The datasets generated in this study are available on request to Abhishek Mahajan (drabhishek.mahajan@yahoo.in).

### Copyright

© The Author(s) 2023.
