# Correction: Deep learning based automated epidermal growth factor receptor and anaplastic lymphoma kinase status prediction of brain metastasis in non-small cell lung cancer

**DOI:** 10.37349/etat.2024.00248

**Published:** 2024-07-08

**Authors:** Abhishek Mahajan, Gurukrishna B, Shweta Wadhwa, Ujjwal Agarwal, Ujjwal Baid, Sanjay Talbar, Amit Kumar Janu, Vijay Patil, Vanita Noronha, Naveen Mummudi, Anil Tibdewal, JP Agarwal, Subhash Yadav, Rajiv Kumar Kaushal, Ameya Puranik, Nilendu Purandare, Kumar Prabhash

**Affiliations:** ^1^Clatterbridge Centre for Oncology NHS Foundation Trust, L7 8YA Liverpool, UK; ^2^Department of Radiodiagnosis, Tata Memorial Hospital, Parel, Mumbai 400012, Maharashtra, India; ^3^Department of Electronics and Telecommunication Engineering, SGGS Institute of Engineering and Technology, Nanded 431606, Maharashtra, India; ^4^Department of Medical Oncology, Tata Memorial Hospital, Parel, Mumbai 400012, Maharashtra, India; ^5^Department of Radiation Oncology, Tata Memorial Hospital, Parel, Mumbai 400012, Maharashtra, India; ^6^Department of Pathology, Tata Memorial Hospital, Parel, Mumbai 400012, Maharashtra, India; ^7^Department of Nuclear Medicine, Tata Memorial Hospital, Parel, Mumbai 400012, Maharashtra, India

In the original published version of the article, the name of author ‘Subhash Yadav’ was incorrectly spelled as ‘Subash Yadav’. This has now been amended in the HTML and PDF versions of the article.

The original uncorrected PDF can be accessed from https://www.explorationpub.com/uploads/er1002158.pdf

