# Supplementary material for: Deep learning based automated epidermal growth factor receptor and anaplastic lymphoma kinase status prediction of brain metastasis in non-small cell lung cancer
Source: Explor Target Antitumor Ther. 2023 Aug 30;4(4):657–68. doi: 10.37349/etat.2023.00158 (PMC10511818; doi:10.37349/etat.2023.00158)
Supplement: Supplementary file 1 [file 1002158_sup_1.pdf]

Table S1. MRI semantic features used in the study

Radiological parameters	MRI features
Site	a. Supratentorial<br>b. Infratentorial<br>c. Both
Depth	a. Subcortical<br>b. Deep<br>c. Both
Side	a. Left<br>b. Right<br>c. Bilateral
Consistency	a. Solid-intermediate signal on T1W and low signal on T2W. Isointense on FLAIR.<br>b. Cystic-low T1W and high T2W signal; loses signal on the FLAIR sequence.<br>c. Mixed-admixture of both.
T1W morphology	a. Hyperintense<br>b. Hypointense<br>c. Isointense
T2W morphology	a. Hyperintense<br>b. Hypointense<br>c. Heterogeneous
T2W borders	a. Fuzzy borders-irregular margins.

	b. Defined borders-smooth margins.
Edema and mass effect	a. None<br>b. Mild<br>c. Moderate<br>d. Significant
Hemorrhage and necrosis	a. Present-tumor has an area of internal high or isolated T1 and low T2; area of internal high T1 and high T2; area of internal low T1 and low T2. Takes into account that the signal characteristics differ depending on the age of the hemorrhage. SWI sequences were also used.<br>b. Absent
Grade of necrosis	a. None<br>b. < 25%<br>c. 25–50%<br>d. > 50%
Enhancement pattern	a. Ring-thick enhancing rim on post-gadolinium T1W.<br>b. Patchy-focal areas of contrast enhancement within the tumour.<br>c. Homogeneous enhancement within tumour.
Diffusion restriction	a. Complete-homogeneous restricted diffusion.<br>b. Central-predominantly in central solid part of lesion.

	c. Peripheral-hyperintense signal of the peripheral solid rim.<br><br>d. None-no areas of restricted diffusion.
Meningeal involvement	a. Present-enhancement of the leptomeninges, small enhancing nodules/seeding along the pia/arachnoid.<br><br>b. Absent
